# Cutaneous Manifestations in a Patient With Reactive Hemophagocytic Lymphohistiocytosis

**DOI:** 10.7759/cureus.10089

**Published:** 2020-08-27

**Authors:** Mohsen Dourra, Shiab Mussad, Robert Singer

**Affiliations:** 1 Medicine, Michigan State University, East Lansing, USA; 2 Dermatology, Ascension Providence Hospital Southfield Campus, Southfield, USA

**Keywords:** hemophagocytic lymphohistiocytosis (hlh), secondary hlh, reactive hlh, rhlh

## Abstract

Hemophagocytic lymphohistiocytosis (HLH) is a rare hematologic disease caused by a disordered immune system. We present a case of reactive HLH (RHLH) with uncommon skin findings in a 35-year-old African American female with a history of hidradenitis suppurativa and morbid obesity. Skin findings on physical exam revealed bullous, ecchymotic, hypopigmented, and ulcerated lesions. The literature on the cutaneous lesions of RHLH is limited. Skin findings are an underrecognized feature of RHLH and can help alert to its presence or its recurrence after treatment.

## Introduction

Hemophagocytic lymphohistiocytosis (HLH) is an aggressive immune-mediated disease with a yearly incidence of 1.2 cases in 1,000,000 people [1]. Manifestations of the disease are a result of excessive cytokine release and improperly activated macrophages, natural killer (NK) cells, and cytotoxic T-cells. Two forms of this systemic disease are described in the literature: the primary (familial) form and the secondary (reactive) form. The reactive form can be due to a myriad of triggers, most commonly infection, lymphoma, and autoimmunity [2]. HLH is associated with high morbidity and mortality. The median survival is two to three months without prompt diagnosis and treatment [3]. 

## Case presentation

**Figure 1:**
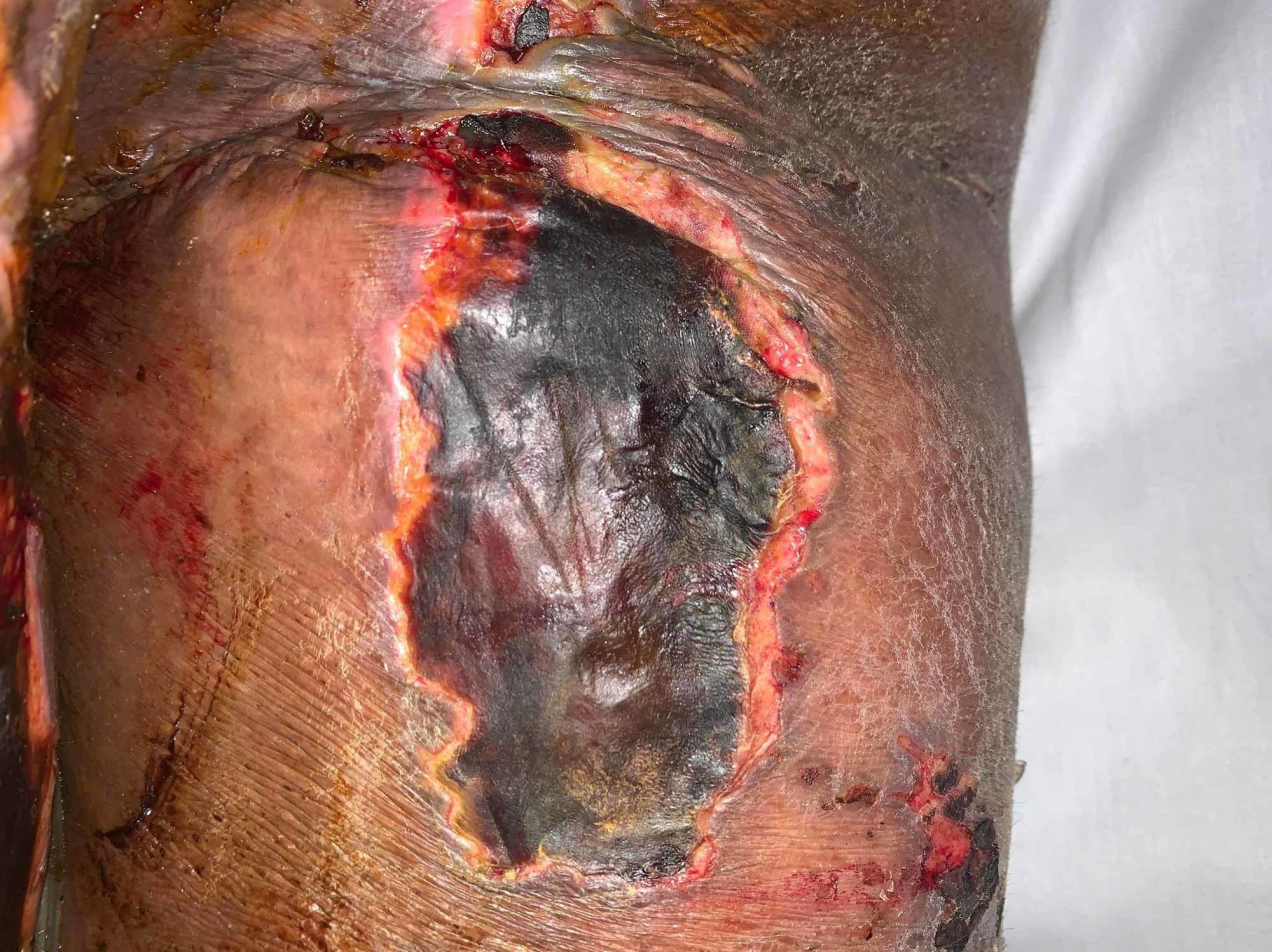
Ulcerated lesion with overlying crusted eschar involving the posterior right thigh.

A 35-year-old African American female with a history of hidradenitis suppurativa initially presented with five days of abdominal pain, nausea and vomiting, and generalized weakness. She also had left lower extremity pain with associated skin lesions. She was afebrile but tachycardic and hypotensive when she arrived at the emergency department with a heart rate of 142 beats/min and a blood pressure of 83/55 mmHg. Physical examination showed signs of dehydration and moderate distress. There was diffuse abdominal tenderness but no signs of peritonitis. Splenomegaly was present. Her critical condition necessitated admission to the intensive care unit. The patient developed what initially appeared to be signs of septic shock, which required vasopressors. She had multiorgan failure that warranted ventilatory support and intubation for 13 days. During that period, she developed diffuse and worsening cutaneous lesions. Skin examination showed tense fluid-filled bullous lesions and superficial ulcerated patches with underlying slough on her medial thighs (Figure 1), sacrum, and lips.

**Figure 2:**
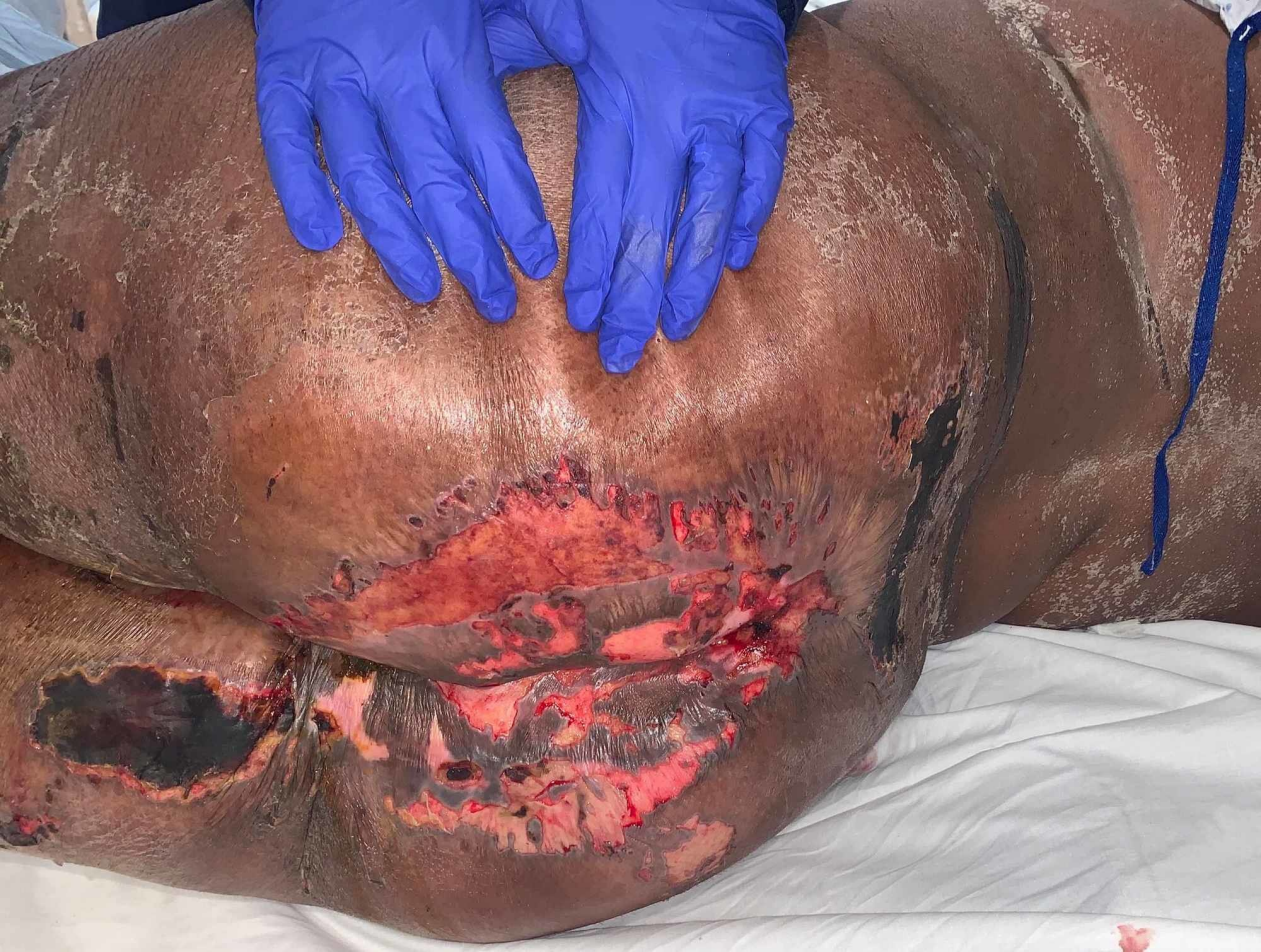
: Flaccid ulcerations due to skin breakdown. Hypopigmentation can be appreciated along the left midback region.

Bullous lesions on the patient’s sacrum developed into desquamating ulcerated plaques (Figure 2). Two annular lesions were found above the patient’s right breast. Multiple superficial ulcerations with underlying hypopigmentation were found above the patent’s left flank and the inferior region of the right breast. Multiple ecchymotic skin lesions were found along the upper and lower extremities bilaterally.

Investigations

Initial labs showed a white blood cell (WBC) count of 8.15 K/mcL (normal: 4-11 K/mcL) and 26% bands. Platelets were decreased at 133,000 K/mcL (normal: 150-400 K/mcL). She had a high anion gap metabolic acidosis at 28 and low bicarbonate at 12 mmol/L (normal: 22-29 mmol/L). Lactic acid was elevated at 8.3 mmol/L (normal: 0.5-2.0 mmol/L). There was mild transaminitis with aspartate aminotransferase at 45 unit/L (normal: 10-35 mmol/L) and alanine aminotransferase at 48 unit/L (normal: 10-35 mmol/L). She had an acute kidney injury with blood urea nitrogen at 45 mg/dL (normal: 6-20 mg/dL) and creatinine at 5.2 mg/dL (normal: 0.5-1.0 mg/dL). Testing for salicylate, ethanol, and acetaminophen was negative. The patient’s WBCs peaked at 57,360 K/mcL and platelets reached a low of 20,000 K/mcL. After admission, an exhaustive workup was performed to search for underlying causes as her clinical status continued to rapidly deteriorate. Blood and tissue cultures had no growth throughout her hospital stay. Testing for infectious agents, including cytomegalovirus, Epstein-Barr virus, and HIV, was negative. Testing for autoimmune etiologies was equally exhaustive and did not yield an underlying disorder. Skin changes secondary to medications were ruled out. 

Ultrasound of the sacral region and left medial thigh, where she had notable skin lesions, showed subcutaneous edema. CT of the extremity did not show any evidence of necrotizing infection. A 3-mm punch biopsy was obtained from the left medial thigh and sent for pathology. It showed epidermal necrosis, superficial dermal necrosis, and necrosis of eccrine coils. There was also prominent dermal hemorrhage and diffuse interstitial neutrophilic infiltrate. Rare vessels in the deep dermis showed fibrinoid necrosis within the vessel wall with some angiocentric neutrophils.

**Figure 3:**
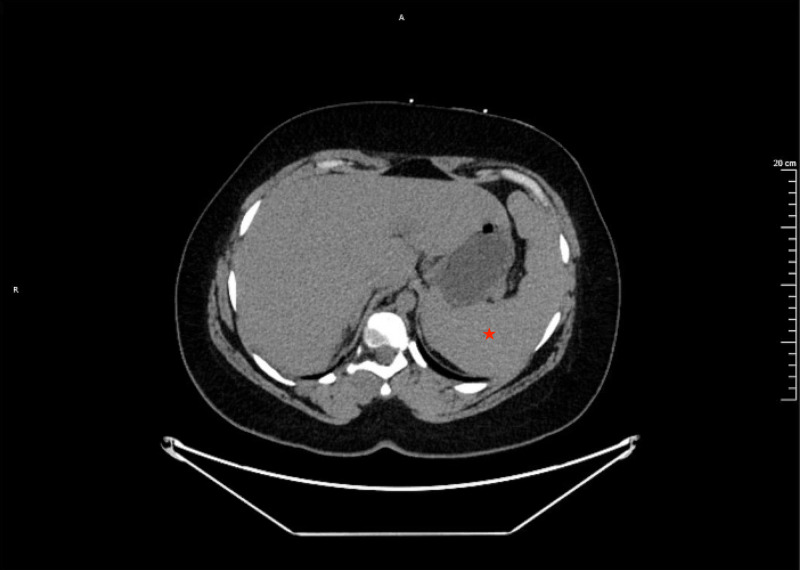
CT of the abdomen showing prominent splenomegaly measuring 15.9 cm (normal: ≤12 cm) along the anteroposterior diameter.

Hematology and oncology services were consulted, and a diagnosis of reactive HLH (RHLH) was ultimately determined based on the presence of five of eight criteria required for diagnosis. The patient’s ferritin was elevated at 2,053 ng/mL (normal: 13-150 ng/mL), triglycerides were elevated at 1,658 mg/dL (normal: <150 mg/dL), platelets were low at 20,000 K/mcL (normal: 150-400 K/mcL), hemoglobin was low at 7.3 gm/dL (normal: 12-16 gm/dL), and soluble CD25, measured via sternal bone marrow biopsy, was elevated at 12,000 U/mL (normal: ≤1,033 U/mL). Bone marrow biopsy did not show evidence of infection or malignancy, and hemophagocytosis was not observed. On initial physical examination, splenomegaly was appreciated. This was confirmed on CT of the abdomen and pelvis, which showed evidence of a prominent spleen (Figure 3) measuring 15.9 cm (normal: ≤12 cm) in the anteroposterior diameter without signs of any lesions.

Outcome

The patient was in critical condition after developing multiorgan failure, which required intubation for ventilatory support. Septic shock was initially suspected as a possible underlying trigger, but an infectious disease workup did not yield a cause. The patient was started on steroids and began showing signs of improvement, including improvement of her WBC count and oxygenation. After meeting criteria for RHLH, she was started on a regimen of etoposide, while continuing steroids. The patient showed remarkable clinical improvement after her very first dose of etoposide. She became awake and alert, and she was able to move all four extremities. There was improvement of her blistering skin lesions with decreased associated pain, as well. However, the patient continued to have a protracted clinical course and was transferred to a tertiary care center for continued management. She was eventually discharged home after recovering. Her skin manifestations resolved. 

## Discussion

HLH occurs as a result of excessive immune stimulation that leads to widespread tissue inflammation and injury. A multifactorial etiology involving defects in macrophages, NK cells, and cytotoxic T-cells underlies this process [4]. There is a systemic elevation of cytokines, including soluble interleukin-2 receptor (sIL-2R), interferon γ, interleukin 2, interleukin 12, and other inflammatory markers. Increased levels of sIL-2R, also known as soluble CD25, can be found in patients with RHLH and is used as a reliable indicator of disease presence and severity. Levels of sIL-2R ≤2400 U/mL have a sensitivity of 100% and levels >10,000 U/mL have a specificity of 93%, according to a retrospective study of 78 adults who had sIL-2R measured for suspected HLH [5]. Our patient had a sIL-2R of 12,000 U/mL, which was measured via sternal bone marrow biopsy.

HLH triggers are typically categorized as primary or secondary. The primary or familial form is the result of inherited gene mutations in immune regulation. The secondary or reactive form occurs when dysfunction is due to an acquired source, such as infection, malignancy, or an autoimmune process. Adults often develop HLH in the background of serious disease making diagnosis a challenging endeavor, especially when the clinical picture appears similar to sepsis. This was the case with our patient who had multiorgan failure without an underlying infectious process. The most common clinical signs of HLH include fever, splenomegaly, hepatomegaly, pulmonary involvement, peripheral adenopathy, and skin lesions. Skin findings can lead to the inclusion of HLH in the differential diagnosis [2].

The diagnosis of RHLH can be determined molecularly or with the utilization of clinical and laboratory criteria. These diagnostic guidelines were created by the histiocyte society in 1991 and were updated in 2004. Diagnosis can be made if patients meet five out of the following eight criteria: fever, splenomegaly, bicytopenia, hypertriglyceridemia (fasting >265 mg/dL) and/or hypofibrinogenemia (<150 mg/dL), hemophagocytosis in the absence of malignancy, low/absent NK cell activity, hyperferritinemia (>500 ng/mL), and increased sIL-2R (>2,400 U/mL) [5,6]. Our patient met five of the eight criteria required for diagnosis. She exhibited clinical and CT-correlated splenomegaly, bicytopenia (anemia and thrombocytopenia), hypertriglyceridemia, hyperferritinemia, and elevated sIL-2R levels.

In the few studies on RHLH, cutaneous findings were reported in 10%-20% of cases. These findings were largely described as non-specific. One retrospective study by Fardet et al. detailed three different categories of skin manifestations observed in RHLH patients. Categories were defined as (1) specific to the underlying cause of disease such as lymphoma or infection, (2) a generalized maculopapular rash, and (3) a result of excess cytokine release (e.g. thrombocytopenic purpura). The most common presentation in that study was a generalized non-pruritic maculopapular rash. Mucosal lesions, hypopigmentation, and epidermal changes were not observed by Fardet et al. [7]. Our patient developed those findings, including a unique presentation of bullous lesions at various stages of healing. To the best of our knowledge, no previous reports have detailed a similar cutaneous presentation.

Notably, skin findings can also signify the presence of an underlying T-cell lymphoma. A close association exists between lymphoma and the presence of subcutaneous, panniculitis-like nodules [2]. This illustrates the importance of recognizing different cutaneous presentations of RHLH since it could help with establishing the underlying source.

Skin biopsy has been investigated as a potential diagnostic tool for HLH [8]. Hemophagocytosis is the histopathological hallmark but is rarely observed in skin biopsy specimens and it was not observed in this case [9,10]. However, we did find evidence on skin biopsy of vasculitis with epidermal necrosis, hemorrhage, and interstitial neutrophilic inflammation. Improvement of skin lesions with chemotherapy and corticosteroids has been reported in several studies [1,4,6]. Our patient had a remarkable response to etoposide and steroids with the eventual resolution of her skin lesions.

## Conclusions

Cutaneous lesions are important findings in patients with RHLH. Skin findings may be apparent at presentation or they may develop during the course of disease. Redevelopment of skin lesions after treatment with chemotherapy may indicate recurrence of HLH. Awareness of these associations may assist with diagnosis and prompt treatment.
